# Macrophage morphology and distribution are strong predictors of prognosis in resected colorectal liver metastases: results from an external retrospective observational study

**DOI:** 10.1097/JS9.0000000000000374

**Published:** 2023-04-12

**Authors:** Guido Costa, Carlo Sposito, Cristiana Soldani, Michela A. Polidoro, Barbara Franceschini, Federica Marchesi, Faizan D. Nasir, Matteo Virdis, Andrea Vingiani, Ana Leo, Luca Di Tommaso, Soumya Kotha, Alberto Mantovani, Vincenzo Mazzaferro, Matteo Donadon, Guido Torzilli

**Affiliations:** a Department of Biomedical Science, Humanitas University, Pieve Emanuele, Milan; b Department of Biotechnology and Translational Medicine; c Department of Oncology and Hemato-Oncology, University of Milan; d Department of Pathology; e Department of Surgery, HPB Surgery and Liver Transplant Unit, Istituto Nazionale Tumori Fondazione IRCCS, Milan; f Department of Hepatobiliary and General Surgery; g Department of Immunology and Inflammation; h Hepatobiliary Immunopathology Unit; i Division of Internal Medicine and Hepatology, Department of Gastroenterology; j Department of Pathology, IRCCS Humanitas Research Hospital, Rozzano, Milan; k Department of Surgery, University Maggiore Hospital della Carità; l Department of Health Sciences, Università del Piemonte Orientale, Novara, Italy; m William Harvey Research Institute, Queen Mary University, London, UK

**Keywords:** colorectal cancer, colorectal liver metastases, hepatectomy, hepatic resection, immune contexture, tumor-associated macrophage

## Abstract

Tumor-associated macrophages (TAMs) are key components of a tumoral microenvironment and have been shown to impact prognosis in different cancers. Previously reported data showed that TAM morphology correlates with prognosis in colorectal liver metastases (CLMs) after hepatectomy, with smaller TAMs (S-TAMs) conferring a more favorable prognosis than larger ones (L-TAMs). This study aims to externally validate this finding.

**Material and methods:**

The external cohort consisted of 84 formalin-fixed and paraffin-embedded surgical samples of CLMs and peritumoral tissue. Two-micrometer-section slides were obtained; the area and perimeter of 21 macrophages in each slide were recorded. The endpoints were TAMs morphometrics and their prognostic significance in relation to disease-free survival (DFS).

**Results:**

The average macrophage perimeter was 71.5±14.1 μm whilst the average area was 217.7±67.8 μm^2^. At univariate analysis, the TAM area demonstrated a statistically significant association with DFS (*P*=0.0006). Optimal area cutoff value was obtained, showing a sensitivity and specificity of 92 and 56%, respectively. S-TAMs and L-TAMs were associated with 3-year DFS rates of 60 and 8.5%, respectively (*P*<0.001). Multivariate analysis confirmed the predictive role of TAM area for DFS [hazard ratio (HR)=5.03; 95% CI=1.70–14.94; *P*=0.003]. Moreover, in a subset of patients (*n*=12) characterized by unfavorable (*n*=6, recurrence within 3 months) or favorable (*n*=6, no recurrence after 48 months) prognosis, TAMs showed a different distribution: L-TAMs were more abundant and closer to the tumor invasive margin in patients that encountered early recurrence and tended to cluster in foci significantly larger (*P*=0.02).

**Conclusions:**

This external validation confirms that morphometric characterization of TAMs can serve as a simple readout of their diversity and allows to reliably stratify patient outcomes and predict disease recurrence after hepatectomy for CLMs.

## Introduction

Highlights

- Tumor-associated macrophages (TAMs) are key components of tumoral microenvironment with prognostic significance in different cancers.
- TAMs have not been widely analyzed and characterized in patients undergoing hepatectomy for colorectal liver metastases (CLMs).
- Morphometric characterization of TAMs can serve as a readout of their diversity and allow for stratification of patient outcomes and prediction of disease recurrence after hepatectomy for CLMs.

According to the WHO Global Cancer Observatory (GLOBOCAN) 2020 data, colorectal cancer (CRC) is the third most common cancer diagnosed and the second-leading cause of mortality amongst all cancers^1^. Over 1.8 million people worldwide were diagnosed with CRC in 2020, 15–25% of them with synchronous colorectal liver metastases (CLM), and another 20% would develop CLM within 3–5 years from the first diagnosis^2^,^3^.

To date, the standard treatment for CLM is surgical resection that, combined with systemic chemotherapy, has the potential to be curative, projecting a 5-year survival rate of up to 50%^3^,^4^. However, these patients present with heterogeneous clinical outcomes and treatment responsiveness^5^,^6^ so much that the finding of distinct biological features associated with clinical presentations to refine patient’s stratification is one of the unmet needs in the care of CLM patients.

Recently, the research on cells and mediators that populate the tumor microenvironment (TME) has led to promising results toward the clinical endpoint of better patient profiling^7^. It has been reported that metastatic TME is strongly immunosuppressed and facilitates the seeding and growth of cancer cells^8^.

Tumor-associated macrophages (TAMs) are key elements of the TME^9^,^10^ since they prepare the tissue invasion and growth of metastasis, serving as a component of the cancer cell niche at distant sites^11^. Their clinical relevance has been reported in large cohorts of different cancer patients, not only as prognostic factors^12^,^13^ but also as a marker of chemotherapy efficacy^14^–^16^. On this line, we recently showed that TAMs in CLM patients include at least two main subtypes of cells that can be identified and measured by conventional immunohistology^17^. Such cells, operationally named large-TAMS (L-TAMS) and small-TAMS (S-TAMS), according to their differential morphometric features and transcriptional signatures, can serve as a marker of strong prognostic significance in CLM patients^17^.

In this study, we sought to pursue our research with external validation of the prognostic significance of TAMs morphology. We further implemented this validation by analyzing the distribution and density of these two populations within the invasive margin of the tumor.

## Material and methods

### Study design

This is a retrospective observational study designed to externally validate and confirm the role of TAMs morphology as a prognostic factor in patients resected for CLM following our previous observation^17^. The study protocol was in accordance with the ethical guidelines established in the 1975 Declaration of Helsinki and compliant with the procedures of the local ethical committee of both IRCCS Humanitas Research Hospital (Milan, Italy – registration number 282/19) and Fondazione IRCCS Istituto Nazionale Tumori (Milan, Italy). Results were reported according to Strengthening the Reporting of Cohort Studies in Surgery (STROCSS guidelines)^18^, Supplemental Digital Content 1, http://links.lww.com/JS9/A307. The protocol was also submitted to the international clinical trial registry (clinicaltrials.gov – registration number NCT03888638).

### Study endpoint

The primary endpoint of this study was the morphometric analysis of TAMs in patients resected for CLMs. The secondary endpoint was the survival and prognostic analysis in relation to TAMs analysis in resected CLM patients. For this purpose, disease-free survival (DFS) and overall survival (OS) were considered as time-to-event endpoints.

### Patients

Patients’ demographic, clinical, surgical, and histopathological data were assembled in a retrospective database for analysis. Inclusion criteria for the study were: age over 18 years, stable or partial response to neoadjuvant chemotherapy, and histologically proven CLM. Exclusion criteria were a progressive disease, a combination of hepatectomy with radiofrequency or microwave ablation, and nonradical liver resection. All patients were discussed at the local multidisciplinary tumor board, in which, according to the most recent international guidelines, each patient received the most appropriate tailored treatment – including the use of neoadjuvant perioperative systemic therapy as well as the order of resection in case of synchronous colorectal tumors and liver metastases. The preoperative workup included 18-F Fluorodeoxyglucose positron emission tomography, total-body contrast-enhanced computed tomography scan, and Gd-EOB-DTPA(gadolinium-ethoxybenzyl-diethylenetriamine pentaacetic acid)-enhanced MRI, performed no more than 30 days prior to surgery. Postoperative follow-up was performed every 3 months and included patients’ examination, serum oncological markers, and abdominal CT or MRI.

### Slide preparation and staining

Formalin-fixed and paraffin-embedded specimens of CLM and peritumor tissue were provided by the Pathology Department of the Fondazione IRCCS Istituto Nazionale Tumori (Milan, Italy). Two-micrometer thick tissue sections were obtained, deparaffinized by two changes of xylene, hydrated in descending grades of alcohol (100, 96, and 70% ethanol, 10 min at each concentration) to water and rehydrated in phosphate-buffered saline (PBS). Antigen retrieval was performed by heat treatment using an ethylenediaminetetraacetic acid (EDTA) buffer (0.25 mM, pH 8; Dako) in a water bath at 98°C for 20 min. After washing with PBS, endogenous peroxidases were blocked via incubation with a Peroxidase-Blocking Solution (Dako) for 15 min at room temperature, and subsequently, to block nonspecific binding, the slides were incubated with Background Sniper (Biocare Medical) for 20 min. After washing with PBS, the sections were then incubated with the primary antibody anti-human CD163 (10D6 clone, diluted 1:200; Leica Biosystems) overnight at room temperature. The slides were washed twice with PBS and then incubated with the detection system EnVision+System HRP-labelled anti-mouse (Dako) for 1 h at room temperature. Following this, diaminobenzidine tetrahydrochloride (Dako) was used as a chromogen to visualize the positive cells. Nuclei were lightly counterstained with a Haematoxylin solution (Dako). The sections were dehydrated and mounted with a mounting medium (Eukitt)^17^.

### Image analysis

**Figure 1:**
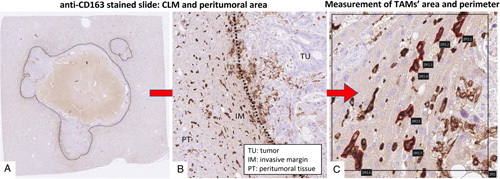
TAMs morphology: assessment. Starting from 2-μm-thick slides stained with the anti-CD163 antibody (A), the metastasis tumor tissue was defined, the zone of the invasive tumor margin in the peritumor tissue was located (B), and the area and perimeter of macrophages were annotated by manually tracing their outlines (C). CLM, colorectal liver metastasis; IM, invasive margin; PT, peritumor tissue; TAMs, tumor-associated macrophages; TU, tumor tissue.

After the staining procedure, slides were digitalized using the Hamamatsu NanoZoomer S360 slide scanner. Image analysis was carried out by the image analysis software Hamamatsu NDP.view2. For each CD163-stained slide, a pathologist, blinded to the study, selected three noncontiguous, nonoverlapping microscopic areas in the invasive margin of the tumor (Fig. 1A, B). In each area, seven randomly selected macrophages were analyzed, and for each cell, the area and perimeter were calculated by manually tracing cell outlines on digitalized images (Fig. 1C).

In a second step, to provide a more detailed analysis of the distribution of the two TAMs (L-TAMs and S-TAMs), we selected a subset of 12 samples from patients characterized by having a particularly unfavorable prognosis (recurrence within 3 months after surgery, *n*=6) or a particularly favorable one (no recurrence after 48 months from surgery, *n*=6). Using the software QuPath 0.3.2, we then built density maps showing the distribution of L-TAMs and S-TAMs on a 500-μm wide peritumor area localized on the whole contour of the tumor. In these maps, each pixel represented the number of L-TAMs counted within a radius of 500 μm. We then calculated the total area of high-density zones of L-TAMs in each slide, defining them as foci of large macrophages: a threshold of 100 L-TAMs within a radius of 200 μm was adopted.

### Statistical analysis

Continuous variables are presented as a range with a median, and discrete variables are presented as a number and percentage. Variables were analyzed using the *χ* ^2^ test or the Mann–Whitney *U* test where appropriate. Kaplan–Meier curves were used to analyzing differences in OS and DFS and were compared using the Log-rank Mantel-Cox test. Survival was calculated from the day of liver surgery. To reduce the differences between groups that may justify a significant variation in survival, risk-adjusted Cox regression analysis was adopted to identify independent prognostic factors for survival. To avoid model overfitting and to limit its optimum performance, we tailored the statistical model using bootstrap resampling^19^–^22^. The effect size of these variables was reported as hazard ratio (HR) and 95% confidential interval (95% CI), *P* values, and survival plots. A *P* value less than 0.05 was considered significant for all tests. For image analysis, the mean value was calculated from the average of three zones per patient. The receiver operating characteristic (ROC) curve and its associated area under the curve (AUC) were generated for relevant TAM metrics to estimate the discriminatory ability for detecting patient survival. Statistical computations were conducted using Stata 16.0 (StataCorp. 2019, Stata Statistical Software: Release 16, StataCorp LLC, College Station, Texas).

## Results

### Patients

The validation cohort included 84 patients aged 18 years or more that underwent hepatic resection for CLMs between 2016 and 2017. Sixty patients were males, and 24 were females, with a median age of 62 (range 36–78). Forty-seven patients (56%) displayed synchronous presentation, 35 (42%) had bilateral liver involvement; the median number of CLMs was 2 (range 1–28), and the median size was 1.9 cm (range 0–11 cm). Neoadjuvant chemotherapy was administered in 59 patients (70%) and included 5-fluorouracil-based protocols in 36 (43%) patients, oxaliplatin-based protocols in 42 (50%) patients, and irinotecan-based protocols in 33 (39%) patients. Biological agents were added in 51 cases [anti-vascular endothelial growth factor (anti-VEGF) in 18% and anti-epidermal growth factor receptor (EGFR) in 33%]. Among patients with synchronous CLM, 21 had a bowel-first approach, 12 had a liver-first approach, and 14 had a simultaneous approach. Postoperative mortality was nil, and postoperative complications occurred in 19 (23%) patients (Table 1). Disease recurrences occurred in 66 patients (79%): median DFS was 12.8 months (range 0.43–58.6) whilst median OS was 36.8 months (range 4.6–58.6). Notably, this external patients’ cohort was comparable with the institutional cohort used in the previous work^17^, presenting, in fact, similar demographic characteristics, the burden of liver disease, use of neoadjuvant treatments, and survival results.

**Table 1:** Description of the series.

Demographics, tumoral, chemotherapy, and postoperative details	
Gender, M/F, *n* (%)	60 (71)/24 (29)
Age, year, median (range)	62 (36–78)
Size of CLMs (cm), median (range)	1.9 (0–11)
Number of CLMs, median (range)	2 (1–28)
Bilobar disease, *n* (%)	35 (42)
Preoperative CEA, median (range)	4 (0.5–520.6)
Preoperative Ca19-9, median (range)	13.45 (0.6–614.3)
Grading of the primary tumor, G3–4, *n* (%)	13 (15)
Staging of the primary tumor	
T status, T3–4, *n* (%)	64 (76)
N status, N+, *n* (%)	50 (60)
Synchronous presentation, *n* (%)	47 (56)
Site of the primary tumor	
Colon, *n* (%)	59 (70)
Rectum, *n* (%)	27 (32)
Neoadjuvant chemotherapy, *n* (%)	59 (70)
Chemotherapy lines, median (range)	1 (1–2)
Type of chemotherapy, *n* (%)	
5-FU based	36 (43)
Oxaliplatin based	42 (50)
Irinotecan based	33 (39)
+Anti-VEGF	18 (21)
+Anti-EGFR	33 (39)
RAS, mutated, *n* (%)	27 (32)
Order of resection	
Bowel first, *n* (%)	21 (45)
Liver first, *n* (%)	12 (25)
Simultaneous resection, *n* (%)	14 (30)
Overall postoperative complications, *n* (%)	19 (23)
Major complications (Clavien–Dindo 3b or more), *n* (%)	5 (6)
90-day postoperative mortality, *n* (%)	0 (0)

5-FU, fluorouracil; CEA, carcinoembryonic antigen; CLMs, colorectal liver metastases; EGFR, epidermal growth factor receptor; M/F, males/females; RAS, rat sarcoma; VEGF, vascular endothelial growth factor.

### TAMs analysis

**Figure 2:**
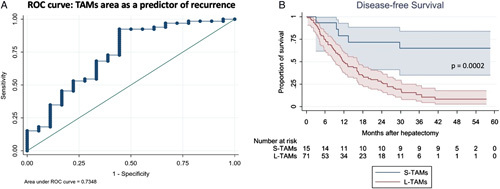
Univariate analysis, the association between TAMs morphology and disease-free survival. A receiver operating characteristic curve was created to define the optimal cutoff point for the prediction of recurrence after hepatectomy (A) and, after univariate analysis (B), macrophage area confirmed a statistically significant association with DFS (*P*=0.0002), with smaller macrophages conferring a more favorable prognosis. DFS, disease-free survival; ROC, receiver operating characteristic; L-TAMs, large TAMs; S-TAMs, small TAMs; TAMs, tumor-associated macrophages.

The TAMs morphometric analysis was computed in all cases. Average TAMs’ perimeter and area were respectively 71.6±14.3 μm and 217.7±68.6 μm^2^. At univariate analysis, the TAM area demonstrated a statistically significant association with DFS. Then, we defined small and large TAMs (S-TAMs and L-TAMs, respectively) using the optimal cutoff value extrapolated from the ROC curve (AUC=0.73 95% CI=0.59–0.88). This resulted in a cutoff area of 151.38 μm^2^ (sensitivity 92%; specificity 56%) (Fig. 2A). Our results showed that S-TAMs and L-TAMs were associated with significantly different 3-year DFS rates of 60 and 8.5%, respectively (*P*=0.0002; Fig. 2B). Univariate analysis was then conducted for age, sex, number of lesions, neoadjuvant chemotherapy, carcinoembryonic antigen (CEA) level, carbohydrate antigen 19-9 (Ca19-9) level, T value of the primary tumor, lymph node metastasis of the primary tumor, synchronous disease, grading of the primary tumor, rat sarcoma (RAS) status, bilobar disease, and site of the primary tumor. Factors that were statistically significant in univariate analysis (Table 2) were then included in a Cox regression multivariate analysis, including the TAM area cutoff value of 151.38 μm^2^. As shown, TAM area (HR=5.03; 95% CI=1.70–14.94; *P*=0.003) was the only statistically significant factor for DFS (Table 3). Finally, the same analysis was approached also using OS as a time-to-event endpoint. Whilst the TAM area maintained its statistical significance in association with OS (*P*=0.034), the association was weaker than with DFS and was not significant in multivariate analysis (Table S1, Supplemental Digital Content 2, http://links.lww.com/JS9/A392).

**Table 2:** Univariate analysis.

	No recurrence	Recurrence	*P*
*n* (%)	18 (28)	66 (79)	
Age, years, median (range)	58.5 (50–70)	62 (36–78)	ns
Sex, male, *n* (%)	12 (60)	49 (74)	ns
Tumor number, *n*, median (range)	1 (1–28)	2 (1–23)	ns
Max size, mm, median (range)	17 (1–45)	20 (1–110)	ns
Neoadjuvant chemotherapy, *n* (%)	8 (44)	51 (77)	<0.01
CEA elevated, *n* (%)	11 (61)	42 (64)	ns
Ca19-9 elevated, *n* (%)	1 (6)	13 (20)	ns
T3–4, *n* (%)	12 (67)	52 (79)	ns
N+, *n* (%)	5 (28)	45 (68)	<0.01
Synchronous presentation, *n* (%)	6 (33)	41 (62)	0.03
G3–4, *n* (%)	2 (11)	11 (17)	ns
KRAS, mutated, *n* (%)	2 (11)	23 (35)	ns
Bilobar disease, *n* (%)	7 (39)	27 (41)	ns
Primary site, rectum, *n* (%)	5 (28)	22 (33)	ns
TAM area	150.4 (97.1–301.1)	217.0 (98.5–388.6)	<0.01

CEA, carcinoembryonic antigen; Ca19-9, carbohydrate antigen 19-9; KRAS, Kirsten rat sarcoma virus; ns, not significant; TAM, tumor-associated macrophages.

**Table 3:** Multivariate analysis.

	Cox model		
Factor	HR	95% CI	*P*
TAMs area; L-TAMs vs. S-TAMs	5.04	1.70–14.94	0.003
Neoadjuvant chemotherapy, yes vs. no	1.27	0.68–2.37	0.453
Number of the primary tumor, positive vs. negative	1.46	0.84–2.55	0.181
Synchronous presentation yes vs. no	1.08	0.63–1.84	0.786

HR, hazard ratio; L-TAMs, large TAMs; S-TAMs, small TAMs; TAMs, tumor-associated macrophages

### Macrophages distribution by density maps

**Figure 3:**
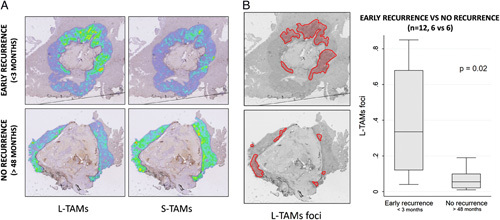
Density maps of TAMs. (A) Density maps showing the distribution of S-TAMs and L-TAMs on a 500-μm wide peritumor area that runs on the whole contour of the tumor in a subset of 12 patients classified as ‘early recurrence’ (recurrence within 3 months after surgery) or ‘no recurrence’ (no recurrence after 48 months from surgery). (B) Histogram showing the total area of the high-density zones of L-TAMs in each slide (defined foci of L-TAMs.) in the two groups of patients (*P*=0.02). S-TAMs, small TAMs; L-TAMs, large TAMs; TAMs, tumor-associated macrophages.

The distribution of macrophages is a critical factor that can regulate their function in the microenvironment and inform on their functional orientation^23^. We wondered whether distribution in the tissue, besides morphology, could be a distinctive feature of L-TAMs. To this aim, we built density maps representing the density of macrophages within a defined radius (500 μm). This distance was chosen to provide an adequate evaluation of the peritumoral invasive margin. As for the previous image analysis, we were able to build the maps in all cases, and the two populations of macrophages showed an interesting different distribution in the two groups of patients. Indeed, those patients who experienced early disease recurrence (within 3 months after hepatectomy) showed a high density of large macrophages (L-TAMs) in the peritumoral tissue, while those patients who experienced late disease recurrence (after 48 months after hepatectomy) showed a higher density of smaller macrophages (S-TAMs) (Fig. 3A). Prompted by these results, suggesting that the aggregation of L-TAMs may be a critical prognostic factor, we analyzed the foci of L-TAMs aggregations in these two groups of patients finding that the early disease recurrence patients’ group had statistically significant larger foci of L-TAMs in the peritumor tissue (*P*=0.02, Fig. 3B).

## Discussion

The TME critically impacts the outcome and therapeutic response in varying cancer subtypes^24^. The composition and magnitude of CD3+ and CD8+ T cells have been linked to a prognostic significance in patients affected by CRC, leading to the successful introduction of an Immunoscore to further generate detailed and personalized patient prognostic information^25^.

On the innate side of the immune system, macrophages, major components of the TME, have been implicated in cancer progression across multiple cancer types^14^,^26^,^27^. Consistently, their clinical relevance has been investigated in large cohorts of cancer patients, confirming a prominent role as prognostic indicators of cancer progression^14^,^26^,^27^. Notable exceptions exist, though, such as CRC^12^ and the therapeutic regimen. Indeed, TAM prognostic value can vary according to the systemic therapy used, which becomes particularly relevant in the metastatic setting because patients undergoing surgery frequently receive more than one line of systemic treatment. Finally, prognostic studies have relied on immunohistochemical analysis of the whole TAM population, while recent multidimensional approaches have shed light on the heterogeneity of TAMs beyond the conventional classification. These considerations raised the question of whether we should try to identify TAM features associated with distinct populations. On these premises, in a previous work, we analyzed TAM morphology in CLMs as a novel feature characteristic of distinct TAM populations and evaluated whether their infiltration can hold prognostic value. Our results demonstrated that although the density of TAMs did not correlate with the survival of CLM patients, cell area was significantly associated with survival, confirming our hypothesis that only a fraction (L-TAMs or S-TAMs) of the whole TAM population have prognostic significance. In detail, patients with small macrophages (S-TAMs) demonstrated 5-year DFS rates of 27.8%, whilst those with large macrophages (L-TAMs) had 5-year DFS of only 0.2%^17^. In the present study, we conducted an external validation of tumor-associated macrophage morphology and its validity as a prognostic model in resected CLMs. Taking profit from an external monocentric series of 84 patients that underwent liver resection for CLMs, we were able to confirm the prognostic role of macrophage morphology. TAMs area demonstrated a statistically significant association with DFS (*P*=0.0006) and was associated with 3-year DFS rates of 60 and 8.5%, respectively (*P*<0.001).

To increase our understanding of the association between TAM area and survival in a subset of patients, we were able to build a map of the density of TAMs in the peritumor tissue. L-TAMs were more abundant and closer to the tumor invasive margin in patients that encountered early recurrence, and foci of L-TAMs were significantly more extended in patients showing a less favorable prognosis. The evidence that S-TAMs and L-TAMs tend to cluster together suggests that morphology implicates distinct macrophage clusters^17^. This was clearly detailed and objectivized with the introduction of the density maps that showed that L-TAMs were more frequently present and closer to the invasive margin of the tumor in patients with worse prognosis and were organized in a cluster, the herein-called *foci*, which were more abundant in those patients.

Our results demonstrate that TAM morphology maintains prognostic significance and is valid as a model to predict disease recurrence after resection for CLMs. This is relevant in a disease like CLMs, which not only experiences a high level of clinical heterogeneity and responsiveness to treatments but is even associated with a relevant recurrence rate after surgical resection^28^. In this sense, it is of paramount importance to identify novel immune classifiers and elucidate the mechanisms behind their prognostic value. For instance, it is known that phagocytic macrophages display high intracellular complexity, with a frequent presentation of intracellular vacuoles, which could signify activation of phagolysosomes or lipid accumulation typically seen in foamy cells^29^.

The present study has some limitations. Despite the fact that we have confirmed our preliminary findings on an external series, the prognostic role of TAMs morphology still requires to be validated by further independent studies. Due to the retrospective nature of this study, the results may be affected by biases in retrieving complete information about patients. Additionally, due to the low number of cases, we could not exclude the risk of type-II error. Moreover, the segmentation of the macrophages is a time-consuming procedure that needs automatization or semi-automatization to be fully incorporated into the workflow of the routine histopathological evaluation of CLM patients. At the same time, the present study has the strength of highlighting the benefits of such translational research that showed how TAMs might be involved in the complex clinical heterogeneity and prognosis of CLM patients.

In conclusion, this external validation study confirmed that quantitative morphometric characterization of TAMs can serve as an easily quantifiable correlate of functional diversity with strong prognostic significance. TAMs can be used to reliably stratify patient outcomes and predict recurrence, with S-TAMs and L-TAMs conferring 3-year DFS of 60% and 8.5%, respectively (*P*=0.0002). These results are consistent with those reported in our previous experience (5-year DFS rates of 27.8 and 0.2%, respectively). Future perspectives could include the development of an AI-based software that can accurately analyze macrophages and output patient outcomes and the development of an internationally validated TAMs-based immunoscore for CLMs. Potential new treatment strategies could aim to specifically target the macrophage compound, including complement components, scavenger and phagocytic receptors, and lipid metabolism to preferentially impact L-TAMs, translating them from a strong prognostic indicator to a therapeutic target.

## Ethical approval

The study protocol was in accordance with the ethical guidelines established in the 1975 Declaration of Helsinki and compliant with the procedures of the local ethical committee of IRCCS Humanitas Research Hospital (Milan, Italy – registration number 282/19).

## Sources of funding

This work was supported by ‘Bando Ricerca Finalizzata 2018 of the Italian Ministry of Health’ (ID=RF-2018-12367150; Principal Investigator = Matteo Donadon). The funding agency had no role in the design of the study or collection and analysis of data.

## Author contribution

M.D. and G.T.: conceptualization; G.C., C.S., F.D.N., M.V., A.V., and S.K.: data curation; G.C., C.S., M.A.P., B.F., F.M., and M.D.: formal analysis; M.D.: funding acquisition; G.C., F.M., A.L.N., and M.D.: investigation; G.C., C.S., F.M., and M.D.: methodology; M.D. and C.S.: project administration; G.C., F.M., A.L.N., S.K., and L.D.T.: software; M.D., G.T., A.M., and V.M.: supervision; G.C., C.S., M.A.P., B.F., and M.D.: visualization; G.C. and M.D.: writing – original draft; M.D., G.T., G.C., C.S., V.M., and A.M.: writing – review and editing.

## Conflicts of interest disclosure

There are no conflicts of interest.

## Research registration unique identifying number (UIN)

1. Name of the registry: clinicaltrials.gov.
2. Unique identifying number or registration ID: NCT03888638.
3. Hyperlink to your specific registration (must be publicly accessible and will be checked): https://clinicaltrials.gov/ct2/show/NCT03888638

## Guarantor

All the authors serve as guarantors.

## Data availability statement

Data is available on a reasonable request to the corresponding author.

## Provenance and peer review

Not commissioned, externally peer-reviewed.

## Supplementary Material
