# Supplementary material for: Macrophage morphology and distribution are strong predictors of prognosis in resected colorectal liver metastases: results from an external retrospective observational study
Source: Int J Surg. 2023 Apr 12;109(5):1311–7. doi: 10.1097/JS9.0000000000000374 (PMC10389408; doi:10.1097/JS9.0000000000000374)
Supplement: Supplementary file 2 [file js9-109-1311-s002.docx]

**Title**

Macrophage morphology and distribution are strong predictors of prognosis in resected colorectal liver metastases: results from an external retrospective observational study

clinicaltrials.gov – registration number NCT03888638

**Authors**

Guido Costa^1,2^*, Carlo Sposito^4,5^ *, Cristiana Soldani^3^, Michela Anna Polidoro^3^, Barbara Franceschini^3^, Federica Marchesi^6,7^, Faizan Daniyal Nasir^2^, Matteo Virdis^5^, Andrea Vingiani^8^, Ana Lleo de Nalda^1,9^, Luca Di Tommaso^1,10^, Soumya Kotha^10^, Alberto Mantovani^1,7,11^, Vincenzo Mazzaferro^4,5^, Matteo Donadon^12,13‡^, Guido Torzilli^1,2‡^

**Affiliations**

^1^ Department of Biomedical Science, Humanitas University, Pieve Emanuele, Milan, Italy.

^2^ Department of Hepatobiliary and General Surgery, IRCCS Humanitas Research Hospital, Rozzano, Italy.

^3^ Hepatobiliary Immunopathology Unit, IRCCS Humanitas Research Hospital, Rozzano, Italy.

^4^ Department of Oncology and Hemato-Oncology, University of Milan, Italy.

^5^ Department of Surgery, HPB Surgery and Liver Transplant Unit, Istituto Nazionale Tumori Fondazione IRCCS, Milan, Italy.

^6^ Department of Biotechnology and Translational Medicine, University of Milan, Italy.

^7^ Department of Immunology and Inflammation, IRCCS Humanitas Research Hospital, Rozzano, Italy.

^8^ Department of Pathology, Istituto Nazionale Tumori Fondazione IRCCS, Milan, Italy.

^9^ Division of Internal Medicine and Hepatology, Department of Gastroenterology, IRCCS Humanitas Research Hospital, Rozzano, Milan, Italy.

^10^ Department of Pathology, IRCCS Humanitas Research Hospital, Rozzano, Italy.

^11^ William Harvey Research Institute, Queen Mary University, London EC1M 6BQ, UK.

^12^ Department of Health Sciences, Università del Piemonte Orientale, Novara, Italy

^13^ Department of Surgery, University Maggiore Hospital della Carità, Novara, Italy

*Theese authors equally contributed as first authors

‡These authors equally contributed as senior authors.

**Correspondence to**

Matteo Donadon, MD, PhD

Associate Professor of Surgery

Department of Health Sciences, Università del Piemonte Orientale

Via Solaroli 17, 28100, Novara, Italy

Director, Surgical Oncology Program, Department of Surgery

University Maggiore Hospital della Carità

Corso Mazzini 18, 28100, Novara, Italy

Tel: +39-0321-3733045 | E-mail: matteo.donadon@uniupo.it

**Highlights**

- Tumor-associated macrophages (TAMs) are key components of tumoral microenvironment

with prognostic significance in different cancers.

- TAMs have not been widely analyzed and characterize in patients undergoing hepatectomy for colorectal liver metastases (CLMs).
- Morphometric characterization of TAMs can serve as a readout of their diversity and allows to stratify patient outcomes and predict disease recurrence after hepatectomy for CLMs.

**Data statement**

Data available on a reasonable request to the corresponding author.

**Abstract**

Introduction: Tumor-associated macrophages (TAMs) are key components of tumoral microenvironment and have been shown to impact prognosis in different cancers. Previously reported data showed that TAM morphology correlates with prognosis in colorectal liver metastases (CLMs) after hepatectomy, with smaller TAMs (S-TAMs) conferring a more favorable prognosis than larger ones (L-TAMs). This study aims to externally validate this finding.

Methods: The external cohort consisted in 84 formalin-fixed and paraffin-embedded surgical samples of CLMs and peritumoral tissue. Two-μm sections slides were obtained; area and perimeter from 21 macrophages in each slide were recorded. The endpoints were TAMs morphometrics and their prognostic significance in relation to disease-free survival (DFS).

Results: Average macrophage perimeter was 71.5±14.1 μm whilst average area was 217.7±67.8 μm^2^. At univariate analysis, TAM area demonstrated statistically significant association with DFS (P=0.0006). Optimal area cut-off value was obtained, showing a sensitivity and specificity of 92% and 56%, respectively. S-TAMs and L-TAMs were associated with a 3-year DFS rates of 60% and 8.5%, respectively (P<0.001). Multivariate analysis confirmed the predictive role of TAM area for DFS (HR=5.03; 95%CI=1.70-14.94; P=0.003). Moreover, in a subset of patients (n=12) characterized by un-favorable (n=6, recurrence within 3 months) or favorable (n=6, no recurrence after 48 months) prognosis, TAMs showed a different distribution: L-TAMs were more abundant and closer to the tumor invasive margin in patients that encountered early recurrence and tended to cluster in foci significantly larger (P=0.02).

Conclusions: This external validation confirms that morphometric characterization of TAMs can serve as a simple readout of their diversity and allows to reliably stratify patient outcomes and predict disease recurrence after hepatectomy for CLMs.

**Introduction**

According to the World Health Organization (WHO) Global Cancer Observatory (GLOBOCAN) 2020 data, colorectal cancer (CRC) is the third most common cancer diagnosed, and the second-leading cause of mortality amongst all cancers [[1]](https://sciwheel.com/work/citation?ids=10431948&pre=&suf=&sa=0&dbf=0). Over 1.8 million people worldwide have been diagnosed with CRC in 2020, 15 to 25% of them with synchronous colorectal liver metastases (CLM), and with another 20% that would develop CLM within 3-5 years from the first diagnosis [[2,3]](https://sciwheel.com/work/citation?ids=9815488,11146058&pre=&pre=&suf=&suf=&sa=0,0&dbf=0&dbf=0).

To date, the standard treatment for CLM is surgical resection that, combined with systemic chemotherapy, has the potential to be curative projecting the 5-year survival rate up to 50%[[3,4]](https://sciwheel.com/work/citation?ids=11146058,13419746&pre=&pre=&suf=&suf=&sa=0,0&dbf=0&dbf=0). However, these patients present with heterogeneous clinical outcomes and treatment responsiveness [[5,6]](https://sciwheel.com/work/citation?ids=5798191,4294528&pre=&pre=&suf=&suf=&sa=0,0&dbf=0&dbf=0) so much that the finding of distinct biological features associated to clinical presentations to refine patient’s stratification is one of the unmet needs in the care of CLM patients.

Recently, the research on cells and mediators that populate the tumor microenvironment (TME) has led to promising results toward the clinical endpoint of better patient profiling [[7]](https://sciwheel.com/work/citation?ids=3993302&pre=&suf=&sa=0&dbf=0). It has been reported that metastatic TME is strongly immunosuppressed and facilitates the seeding and growth of cancer cells [[8]](https://sciwheel.com/work/citation?ids=11446662&pre=&suf=&sa=0&dbf=0).

Tumor-associated macrophages (TAMs) are key-elements of the TME [[9,10]](https://sciwheel.com/work/citation?ids=75688,93550&pre=&pre=&suf=&suf=&sa=0,0&dbf=0&dbf=0) since they prepare the tissue invasion and growth of metastasis, serving as a component of the cancer cell niche at distant sites [[11]](https://sciwheel.com/work/citation?ids=3332761&pre=&suf=&sa=0&dbf=0). Their clinical relevance has been reported in large cohorts of different cancer patients, not only as prognostic factors[[12,13]](https://sciwheel.com/work/citation?ids=1633307,242936&pre=&pre=&suf=&suf=&sa=0,0&dbf=0&dbf=0) but also as marker of chemotherapy efficacy [[14–16]](https://sciwheel.com/work/citation?ids=13484233,13430282,12106941&pre=&pre=&pre=&suf=&suf=&suf=&sa=0,0,0&dbf=0&dbf=0&dbf=0). On this line, we recently showed that TAMs in CLM patients include at least two main subtypes of cells that can be identified and measured by conventional immunohistology [[17]](https://sciwheel.com/work/citation?ids=9506831&pre=&suf=&sa=0&dbf=0). Such cells, operationally named large-TAMS (L-TAMS) and small-TAMS (S-TAMS) according to their differential morphometric features and transcriptional signatures, can serve as a marker of strong prognostic significance in CLM patients [[17]](https://sciwheel.com/work/citation?ids=9506831&pre=&suf=&sa=0&dbf=0).

In this study, we sought to pursue our research with an external validation of the prognostic significance of TAMs morphology. We further implemented this validation by analyzing the distribution and density of these two populations within the invasive margin of the tumor.

**Material and methods**

*Study design*

The is a retrospective observational study designed to externally validate and confirm the role of TAMs morphology as a prognostic factor in patients resected for CLM following our previous observation [[17]](https://sciwheel.com/work/citation?ids=9506831&pre=&suf=&sa=0&dbf=0). The study protocol was in accordance with the ethical guidelines established in the 1975 Declaration of Helsinki and compliant to the procedures of the local ethical committee of both IRCCS Humanitas Research Hospital (Milan, Italy – registration number 282/19) and Fondazione IRCCS Istituto Nazionale Tumori (Milan, Italy). Results were reported according to Strengthening the Reporting of Cohort Studies in Surgery (STROCSS guidelines) [[18]](https://sciwheel.com/work/citation?ids=11723579&pre=&suf=&sa=0&dbf=0), Supplemental Digital Content 1, <http://links.lww.com/JS9/A307>. The protocol was also submitted to the international clinical trial registry (clinicaltrials.gov – registration number NCT03888638).

*Study endpoint*

The primary endpoint of this study was the morphometric analysis of TAMs in patients resected for CLMs. The secondary endpoint was the survival and prognostic analysis in relation with TAMs analysis in resected CLM patients. For this purpose, disease-free survival (DFS) and overall survival (OS) were considered as time-to-event endpoints.

*Patients*

Patients’ demographic, clinical, surgical, and histopathological data were assembled in a retrospective database for analysis. Inclusion criteria for the study were: age over 18 years, stable or partial response to neoadjuvant chemotherapy, histologically proven CLM. Exclusion criteria were progressive disease, combination of hepatectomy with radiofrequency or microwave ablation, and nonradical liver resection. All patients were discussed at the local multidisciplinary tumor board, in which according to the most recent international guidelines each patient received the most appropriate tailored treatment – including the use of neoadjuvant perioperative systemic therapy as well as the order of resection in case of synchronous colorectal tumors and liver metastases. The preoperative workup included 18-F Fluorodeoxyglucose positron emission tomography, total-body contrast-enhanced CT scan and Gd-EOB-DTPA enhanced MRI, performed no more than 30 days prior to surgery. Postoperative follow up was performed every 3 months and included patients’ examination, serum oncological markers and abdominal CT or MRI.

*Slide preparation and staining*

Formalin-fixed and paraffin-embedded specimen of CLM and peritumor tissue were provided by the Pathology Department of the Fondazione IRCCS Istituto Nazionale Tumori (Milan, Italy). 2-μm thick tissue sections were obtained, deparaffinized by 2 changes of xylene, hydrated in descending grades of alcohol (100, 96 and 70% ethanol, 10 min at each concentration) to water and rehydrated in phosphate-buffered saline (PBS). Antigen retrieval was performed by heat treatment using an ethylenediaminetetraacetic acid (EDTA) buffer (Dako; 0.25 mM, pH 8) in a water bath at 98ºC for 20 minutes. After washing with PBS, endogenous peroxidases were blocked via incubation with a Peroxidase-Blocking Solution (Dako) for 15 minutes at room temperature, and subsequently to block nonspecific binding, the slides were incubated with Background Sniper (BioCare Medical) for 20 minutes. After washing with PBS, the sections were then incubated with the primary antibody anti-human CD163 (Leica Biosystems, 10D6 clone, diluted 1:200) overnight at room temperature. The slides were washed twice with PBS and then incubated with the detection system EnVision+System HRP-labelled anti-mouse (Dako) for 1h at room temperature. Following this, diaminobenzidine tetrahydrochloride (Dako) was used as a chromogen to visualize the positive cells. Nuclei were lightly counterstained with a Haematoxylin solution (Dako). The sections were dehydrated and mounted with a mounting medium (Eukitt) [[17]](https://sciwheel.com/work/citation?ids=9506831&pre=&suf=&sa=0&dbf=0).

*Image Analysis*

After the staining procedure, slides were digitalized using the Hamamatsu NanoZoomer S360 slide scanner. Image analysis was carried out by the image analysis software Hamamatsu NDP.view2. For each CD163-stained slide, a pathologist, blinded to the study, selected three non-contiguous, non-overlapping microscopic areas in the invasive margin of the tumor (Fig.1a,-b). In each area, 7 randomly selected macrophages were analyzed and for each cell, area and perimeter were calculated by manually tracing cell outlines on digitalized images (Fig. 1c).

In a second step, to provide a more detailed analysis of the distribution of the two TAMs (L-TAMs and S-TAMs), we selected a subset of 12 samples, from patients characterized by having a particularly unfavorable prognosis (recurrence within three months after surgery, n=6) or a particularly favorable one (no recurrence after 48 months from surgery, n=6). Using the software QuPath 0.3.2, we then built density maps showing the distribution of L-TAMs and S-TAMs on a 500 μm wide peritumor area localized on the whole contour of the tumor. In these maps, each pixel represented the number of L-TAMs counted within a radius of 500 μm. We then calculated the total area of high-density zones of L-TAMs in each slide defining them *foci* of large macrophages: a threshold of 100 L-TAMs within a radius of 200 μm was adopted.

*Statistical Analysis*

Continuous variables are presented as a range with median, and discrete variables are presented as a number and percentage. Variables were analyzed using the χ^2^ test or the Mann-Whitney U test where appropriate. Kaplan-Meier curves were used to analyze differences in OS and DFS and were compared using the Log-rank Mantel-Cox test. Survival was calculated from the day of liver surgery. To reduce the differences between groups that may justify a significant variation in survival, risk-adjusted Cox regression analysis was adopted to identify independent prognostic factors for survival. To avoid model overfitting and to limit its optimistic performance, we tailored the statistical model using bootstrap resampling [19-22]. The effect size of these variables was reported as hazard ratio (HR) and 95% confidential interval (95%CI), P values, and survival plots. A P value <.05 was considered significant for all tests. For image analysis, the mean value was calculated from the average of three zones per patient. Receiver operating characteristic (ROC) curve and its associated area under the curve (AUC) were generated for relevant TAM metrics to estimate the discriminatory ability for detecting patient survival. Statistical computations were conducted using Stata 16.0 © (StataCorp. 2019. Stata Statistical Software: Release 16. College Station, TX: StataCorp LLC.).

**Results**

*Patients*

The validation cohort included 84 patient aged 18 years or more that underwent hepatic resection for CLMs between 2016 and 2017. Sixty patients were males and 24 females, with a median age of 62 (range 36-78). Forty-seven patients (56%) displayed synchronous presentation, 35 (42%) had bilateral liver involvement; median number of CLMs was 2 (range 1-28) and median size was 1.9 cm (range 0-11cm). Neoadjuvant chemotherapy was administered in 59 patients (70%) and included 5-fluorouracile-based protocols in 36 (43%) patients, oxaliplatin-based protocols in 42 (50%) patients, and irinotecan-based protocols in 33 (39%) patients. Biological agents were added in 51 cases (anti-VEGF in 18%, and anti-EGFR in 33%). Among patients with synchronous CLM, 21 had bowel-first approach, 12 had liver-first approach, and 14 had simultaneous approach. Postoperative mortality was nil, and postoperative complications occurred in 19 (23%) patients (Table 1). Disease recurrences occurred in 66 patients (79%): median DFS was 12.8 months (range 0.43-58.6) whilst median OS was 36.8 months (range 4.6-58.6). Notably, this external patients’ cohort was comparable with the institutional cohort used in the previous work [[17]](https://sciwheel.com/work/citation?ids=9506831&pre=&suf=&sa=0&dbf=0) presenting, in fact, similar demographics characteristics, burden of liver disease, use of neoadjuvant treatments, and survival results.

*TAMs analysis*

TAMs’ morphometric analysis was computed in all cases. Average TAMs’ perimeter and area were respectively 71.6 ± 14.3 μm and 217.7 ± 68.6 μm^2^. At univariate analysis, TAM area demonstrated a statistically significant association with DFS. Then, we defined small and large TAMs (S-TAMs and L-TAMs respectively) using the optimal cut-off value extrapolated from the ROC curve (AUC=0.73 95%CI=0.59-0.88). This resulted in a cut-off area of 151.38 μm^2^ (sensitivity 92%; specificity 56%) (Fig. 2a). Our results showed that S-TAMs and L-TAMs were associated with significantly different 3-year DFS rates of 60% and 8.5% respectively (P=0.0002; Fig. 2b). Univariate analysis was then conducted for age, sex, number of lesions, neoadjuvant chemotherapy, CEA level, Ca19-9 level, T value of the primary tumor, lymph node metastasis of the primary tumor, synchronous disease, grading of primary tumor, RAS status, bilobar disease and site of the primary tumor. Factors that were statistically significant at univariate analysis (Table 2) were then included in a Cox regression multivariate analysis including the TAM area cut-off value of 151.38 μm^2^. As showed, TAM area (HR=5.03; 95%CI=1.70-14.94; P=0.003) was the only statistically significant factor for DFS (Table 3). Finally, the same analysis was approached using also OS as time-to-event endpoint. Whilst TAM area maintained its statistical significance in association with OS (P=0.034), the association was weaker than with DFS, and was not significant at multivariate analysis (Table S1).

*Macrophages distribution by density maps*

Distribution of macrophages is a critical factor that can regulate their function in the microenvironment and inform on their functional orientation [[23]](https://sciwheel.com/work/citation?ids=14041043&pre=&suf=&sa=0&dbf=0). We wondered whether distribution in the tissue, besides morphology, could be a distinctive feature of L-TAMs. To this aim, we built density maps, representing the density of macrophages within a defined radius (500 μm). This distance was chosen to provide an adequate evaluation of the peritumoral invasive margin. As for the previous image analysis, we were able to build the maps in all cases, and the two populations of macrophages showed an interesting different distribution in the two groups of patients. Indeed, those patients who experienced early disease recurrence (within three months after hepatectomy) showed high density of large macrophages (L-TAMs) in the peritumoral tissue, while those patients who experienced late disease recurrence (after 48 months after hepatectomy) showed higher density of smaller macrophages (S-TAMs) (Fig 3a). Prompted by these results, suggesting that the aggregation of L-TAMs may be a critical prognostic factor, we analyzed the foci of L-TAMs aggregations in these two groups of patients finding that the early disease recurrence patients’ group had statistically significant larger foci of L-TAMs in the peritumor tissue (p=0.02, Fig 3b).

**Discussion**

The TME critically impacts on outcome and therapeutic response in varying cancer subtypes [[24]](https://sciwheel.com/work/citation?ids=93186&pre=&suf=&sa=0&dbf=0). The composition and magnitude of CD3+ and CD8+ T cells has been linked to a prognostic significance in patients affected by CRC, leading to the successful introduction of an Immunoscore to further generate detailed and personalized patient prognostic information [[25]](https://sciwheel.com/work/citation?ids=5331249&pre=&suf=&sa=0&dbf=0).

On the innate side of the immune system, macrophages, major components of the TME, have been implicated in cancer progression across multiple cancer types [[14,26,27]](https://sciwheel.com/work/citation?ids=636918,6367684,13484233&pre=&pre=&pre=&suf=&suf=&suf=&sa=0,0,0&dbf=0&dbf=0&dbf=0). Consistently, their clinical relevance has been investigated in large cohorts of cancer patients, confirming a prominent role as prognostic indicators of cancer progression [[14,26,27]](https://sciwheel.com/work/citation?ids=636918,6367684,13484233&pre=&pre=&pre=&suf=&suf=&suf=&sa=0,0,0&dbf=0&dbf=0&dbf=0). Notable exceptions exist, though, such as colorectal cancer [[12]](https://sciwheel.com/work/citation?ids=1633307&pre=&suf=&sa=0&dbf=0) and the therapeutic regimen. Indeed, TAM prognostic value can vary according to the systemic therapy used, which becomes particularly relevant in the metastatic setting because patients undergoing surgery frequently received more than one line of systemic treatment. Finally, prognostic studies have relied on immunohistochemical analysis of the whole TAM population, while recent multidimensional approaches have shed light on the heterogeneity of TAMs beyond the conventional classification. These considerations raised the question whether we should try to identify TAM features associated to distinct populations. On these premises, in a previous work we analyzed TAM morphology in CLMs as a novel feature characteristic of distinct TAM populations and evaluated whether their infiltration can hold prognostic value. Our results demonstrated that although the density of TAMs did not correlate with survival of CLM patients, cell area was significantly associated to survival, confirming our hypothesis that only a fraction (L-TAMs or S-TAMs) of the whole TAM population have prognostic significance. In detail, patients with small macrophages (S-TAMs) demonstrated 5-year disease free survival (DFS) rates of 27.8%, whilst those with large macrophages (L-TAMs) had 5-year DFS of only 0.2% [[17]](https://sciwheel.com/work/citation?ids=9506831&pre=&suf=&sa=0&dbf=0). In the present study, we conducted an external validation of tumor associated macrophage morphology and their validity as a prognostic model in resected CLMs. Taking profit of an external monocentric series of 84 patients that underwent liver resection for CLMs, we were able to confirm the prognostic role of macrophage morphology. TAMs area demonstrated a statistically significant association with DFS (p=0.0006) and was associated with a 3-year DFS rates of 60% and 8.5%, respectively (P<0.001).

To increase our understanding of the association of TAM area and survival, in a subset of patients, we were able to build a map of the density of TAMs in the peritumor tissue. L-TAMs were more abundant and closer to the tumor invasive margin in patients that encountered early recurrence, and foci of L-TAMs were significantly more extended in patients showing a less favorable prognosis. The evidence that S-TAMs and L-TAMs tend to cluster together suggests that morphology implicates distinct macrophage clusters [[17]](https://sciwheel.com/work/citation?ids=9506831&pre=&suf=&sa=0&dbf=0). This was clearly detailed and objectivized with the introduction of the density maps that showed that L-TAMs were more frequently present and closer to the invasive margin of the tumor in patients with worse prognosis and were organized in cluster, the herein called *foci*, that were more abundant in those patients.

Our results demonstrate that TAM morphology maintains prognostic significance and is valid as a model to predict disease recurrence after resection for CLMs. This is relevant in a disease like CLMs, which not only experiences a high-level of clinical heterogeneity and of responsiveness to treatments, but it is even associated to a relevant recurrence rate after surgical resection [[28]](https://sciwheel.com/work/citation?ids=697513&pre=&suf=&sa=0&dbf=0). In this sense, it is of paramount importance to identify novel immune classifiers and elucidate the mechanisms behind their prognostic value. For instance, it is known that phagocytic macrophages display high intracellular complexity, with frequent presentation of intracellular vacuoles, which could signify activation of phagolysosomes or lipid accumulation typically seen in foamy cells [[29]](https://sciwheel.com/work/citation?ids=3528097&pre=&suf=&sa=0&dbf=0).

The present study has some limitations. Despite we have confirmed our preliminary findings on an external series, the prognostic role of TAMs morphology still requires to be validated by further independent studies. Due to the retrospective nature of this study, the results may be affected by biases in retrieving complete information about patients. Additionally, due to the low number of cases, we could not exclude the risk of type-II error. Moreover, the segmentation of the macrophages is a time-consuming procedure that needs automatization or semi-automatization to be fully incorporated in the workflow of the routine histopathological evaluation of CLM patients. At the same time, the present study has the strength of highlighting the benefits of such translational research that showed how TAMs may be involved in the complex clinical heterogeneity and prognosis of CLM patients.

In conclusions, this external validation study confirmed that quantitative morphometric characterization of TAMs can serve as an easily quantifiable correlate of functional diversity with strong prognostic significance. TAMs can be used to reliably stratify patient outcomes and predict recurrence, with S-TAMs and L-TAMs conferring 3-year DFS of 60% and 8.5% respectively (P =0.0002). These results are consistent with those reported in our previous experience (5-yr DFS rates of 27.8% and 0.2%, respectively). Future perspectives could include development of an AI based software that can accurately analyze macrophages and output patient outcomes, and the development of an internationally validated TAMs-based immuno-score for CLMs. Potential new treatment strategies could aim to specifically target the macrophage compound, including complement components, scavenger and phagocytic receptors, and lipid metabolism to preferentially impact L-TAMs, translating them from a strong prognostic indicator to a therapeutic target.

**Acknowledgments**

We thank Dr. Nina Cortese for the support in the analysis of the slides.

**Funding**

This work was supported by “Bando Ricerca Finalizzata 2018 of the Italian Ministry of Health (ID=RF-2018-12367150). The funding agency had no role in the design of the study or collection and analysis of data.

**Provenance and peer review**

Not commissioned, externally peer-reviewed.

**References**

1. [H. Sung, J. Ferlay, R.L. Siegel, M. Laversanne, I. Soerjomataram, A. Jemal, F. Bray, Global cancer statistics 2020: GLOBOCAN estimates of incidence and mortality worldwide for 36 cancers in 185 countries., CA Cancer J. Clin. 71 (2021) 209–249. https://doi.org/10.3322/caac.21660.](https://sciwheel.com/work/bibliography/10431948)
2. [C. Hackl, P. Neumann, M. Gerken, M. Loss, M. Klinkhammer-Schalke, H.J. Schlitt, Treatment of colorectal liver metastases in Germany: a ten-year population-based analysis of 5772 cases of primary colorectal adenocarcinoma., BMC Cancer. 14 (2014) 810. https://doi.org/10.1186/1471-2407-14-810.](https://sciwheel.com/work/bibliography/9815488)
3. [A. Cucchetti, A. Ferrero, M. Cescon, M. Donadon, N. Russolillo, G. Ercolani, G. Stacchini, F. Mazzotti, G. Torzilli, A.D. Pinna, Cure model survival analysis after hepatic resection for colorectal liver metastases., Ann. Surg. Oncol. 22 (2015) 1908–1914. https://doi.org/10.1245/s10434-014-4234-0.](https://sciwheel.com/work/bibliography/11146058)
4. [O.J. Garden, M. Rees, G.J. Poston, D. Mirza, M. Saunders, J. Ledermann, J.N. Primrose, R.W. Parks, Guidelines for resection of colorectal cancer liver metastases., Gut. 55 Suppl 3 (2006) iii1-8. https://doi.org/10.1136/gut.2006.098053.](https://sciwheel.com/work/bibliography/13419746)
5. [N. Halama, S. Michel, M. Kloor, I. Zoernig, A. Benner, A. Spille, T. Pommerencke, D.M. von Knebel, G. Folprecht, B. Luber, N. Feyen, U.M. Martens, P. Beckhove, S. Gnjatic, P. Schirmacher, E. Herpel, J. Weitz, N. Grabe, D. Jaeger, Localization and density of immune cells in the invasive margin of human colorectal cancer liver metastases are prognostic for response to chemotherapy., Cancer Res. 71 (2011) 5670–5677. https://doi.org/10.1158/0008-5472.CAN-11-0268.](https://sciwheel.com/work/bibliography/5798191)
6. [B. Mlecnik, M. Van den Eynde, G. Bindea, S.E. Church, A. Vasaturo, T. Fredriksen, L. Lafontaine, N. Haicheur, F. Marliot, D. Debetancourt, G. Pairet, A. Jouret-Mourin, J.-F. Gigot, C. Hubert, E. Danse, C. Dragean, J. Carrasco, Y. Humblet, V. Valge-Archer, A. Berger, F. Pagès, J.-P. Machiels, J. Galon, Comprehensive intrametastatic immune quantification and major impact of immunoscore on survival., J Natl Cancer Inst. 110 (2018). https://doi.org/10.1093/jnci/djx123.](https://sciwheel.com/work/bibliography/4294528)
7. [W.H. Fridman, L. Zitvogel, C. Sautès-Fridman, G. Kroemer, The immune contexture in cancer prognosis and treatment., Nat. Rev. Clin. Oncol. 14 (2017) 717–734. https://doi.org/10.1038/nrclinonc.2017.101.](https://sciwheel.com/work/bibliography/3993302)
8. [D.I. Tsilimigras, P. Brodt, P.-A. Clavien, R.J. Muschel, M.I. D’Angelica, I. Endo, R.W. Parks, M. Doyle, E. de Santibañes, T.M. Pawlik, Liver metastases., Nat. Rev. Dis. Primers. 7 (2021) 27. https://doi.org/10.1038/s41572-021-00261-6.](https://sciwheel.com/work/bibliography/11446662)
9. [A. Mantovani, P. Allavena, A. Sica, F. Balkwill, Cancer-related inflammation., Nature. 454 (2008) 436–444. https://doi.org/10.1038/nature07205.](https://sciwheel.com/work/bibliography/75688)
10. [B. Ruffell, A. Au, H.S. Rugo, L.J. Esserman, E.S. Hwang, L.M. Coussens, Leukocyte composition of human breast cancer., Proc Natl Acad Sci USA. 109 (2012) 2796–2801. https://doi.org/10.1073/pnas.1104303108.](https://sciwheel.com/work/bibliography/93550)
11. [H. Peinado, H. Zhang, I.R. Matei, B. Costa-Silva, A. Hoshino, G. Rodrigues, B. Psaila, R.N. Kaplan, J.F. Bromberg, Y. Kang, M.J. Bissell, T.R. Cox, A.J. Giaccia, J.T. Erler, S. Hiratsuka, C.M. Ghajar, D. Lyden, Pre-metastatic niches: organ-specific homes for metastases., Nat. Rev. Cancer. 17 (2017) 302–317. https://doi.org/10.1038/nrc.2017.6.](https://sciwheel.com/work/bibliography/3332761)
12. [J. Forssell, A. Oberg, M.L. Henriksson, R. Stenling, A. Jung, R. Palmqvist, High macrophage infiltration along the tumor front correlates with improved survival in colon cancer., Clin. Cancer Res. 13 (2007) 1472–1479. https://doi.org/10.1158/1078-0432.CCR-06-2073.](https://sciwheel.com/work/bibliography/1633307)
13. [C. Steidl, T. Lee, S.P. Shah, P. Farinha, G. Han, T. Nayar, A. Delaney, S.J. Jones, J. Iqbal, D.D. Weisenburger, M.A. Bast, A. Rosenwald, H.-K. Muller-Hermelink, L.M. Rimsza, E. Campo, J. Delabie, R.M. Braziel, J.R. Cook, R.R. Tubbs, E.S. Jaffe, G. Lenz, J.M. Connors, L.M. Staudt, W.C. Chan, R.D. Gascoyne, Tumor-associated macrophages and survival in classic Hodgkin’s lymphoma., N. Engl. J. Med. 362 (2010) 875–885. https://doi.org/10.1056/NEJMoa0905680.](https://sciwheel.com/work/bibliography/242936)
14. [A. Mantovani, P. Allavena, F. Marchesi, C. Garlanda, Macrophages as tools and targets in cancer therapy., Nat. Rev. Drug Discov. 21 (2022) 799–820. https://doi.org/10.1038/s41573-022-00520-5.](https://sciwheel.com/work/bibliography/13484233)
15. [G. Di Caro, N. Cortese, G.F. Castino, F. Grizzi, F. Gavazzi, C. Ridolfi, G. Capretti, R. Mineri, J. Todoric, A. Zerbi, P. Allavena, A. Mantovani, F. Marchesi, Dual prognostic significance of tumour-associated macrophages in human pancreatic adenocarcinoma treated or untreated with chemotherapy., Gut. 65 (2016) 1710–1720. https://doi.org/10.1136/gutjnl-2015-309193.](https://sciwheel.com/work/bibliography/13430282)
16. [A. Malesci, P. Bianchi, G. Celesti, G. Basso, F. Marchesi, F. Grizzi, G. Di Caro, T. Cavalleri, L. Rimassa, R. Palmqvist, A. Lugli, V.H. Koelzer, M. Roncalli, A. Mantovani, S. Ogino, L. Laghi, Tumor-associated macrophages and response to 5-fluorouracil adjuvant therapy in stage III colorectal cancer., Oncoimmunology. 6 (2017) e1342918. https://doi.org/10.1080/2162402X.2017.1342918.](https://sciwheel.com/work/bibliography/12106941)
17. [M. Donadon, G. Torzilli, N. Cortese, C. Soldani, L. Di Tommaso, B. Franceschini, R. Carriero, M. Barbagallo, A. Rigamonti, A. Anselmo, F.S. Colombo, G. Maggi, A. Lleo, J. Cibella, C. Peano, P. Kunderfranco, M. Roncalli, A. Mantovani, F. Marchesi, Macrophage morphology correlates with single-cell diversity and prognosis in colorectal liver metastasis., J. Exp. Med. 217 (2020). https://doi.org/10.1084/jem.20191847.](https://sciwheel.com/work/bibliography/9506831)
18. [R. Agha, A. Abdall-Razak, E. Crossley, N. Dowlut, C. Iosifidis, G. Mathew, STROCSS Group, STROCSS 2019 Guideline: Strengthening the reporting of cohort studies in surgery. Int. J. Surg. 72 (2019) 156–165. https://doi.org/10.1016/j.ijsu.2019.11.002.](https://sciwheel.com/work/bibliography/11723579)
19. Copas JB. Using regression models for prediction: shrinkage and regression to the mean. Stat Methods Med Res. 6 (1997) 167-83. https://doi:10.1177/096228029700600206.
20. Pavlou M, Ambler G, Seaman SR, et al. How to develop a more accurate risk prediction model when there are few events. BMJ. 351 (2015) h3868. https://doi:10.1136/bmj.h3868.
21. Moons KG, Altman DG, Vergouwe Y, Royston P. Prognosis and prognostic re- search: application and impact of prognostic models in clinical practice. BMJ. 4 (2009) b606. https://doi:10.1136/bmj.b606.
22. Kulesa A, Krzywinski M, Blainey P, Altman N. Sampling distributions and the bootstrap. Nat Methods. 2015 Jun;12(6):477-8. https://doi:10.1038/nmeth.3414.
23. [J. Martinek, J. Lin, K.I. Kim, V.G. Wang, T.-C. Wu, M. Chiorazzi, H. Boruchov, A. Gulati, S. Seeniraj, L. Sun, F. Marches, P. Robson, A. Rongvaux, R.A. Flavell, J. George, J.H. Chuang, J. Banchereau, K. Palucka, Transcriptional profiling of macrophages in situ in metastatic melanoma reveals localization-dependent phenotypes and function., Cell Rep. Med. 3 (2022) 100621. https://doi.org/10.1016/j.xcrm.2022.100621.](https://sciwheel.com/work/bibliography/14041043)
24. [D. Hanahan, L.M. Coussens, Accessories to the crime: functions of cells recruited to the tumor microenvironment., Cancer Cell. 21 (2012) 309–322. https://doi.org/10.1016/j.ccr.2012.02.022.](https://sciwheel.com/work/bibliography/93186)
25. [F. Pagès, B. Mlecnik, F. Marliot, G. Bindea, F.-S. Ou, C. Bifulco, A. Lugli, I. Zlobec, T.T. Rau, M.D. Berger, I.D. Nagtegaal, E. Vink-Börger, A. Hartmann, C. Geppert, J. Kolwelter, S. Merkel, R. Grützmann, M. Van den Eynde, A. Jouret-Mourin, A. Kartheuser, D. Léonard, C. Remue, J.Y. Wang, P. Bavi, M.H.A. Roehrl, P.S. Ohashi, L.T. Nguyen, S. Han, H.L. MacGregor, S. Hafezi-Bakhtiari, B.G. Wouters, G.V. Masucci, E.K. Andersson, E. Zavadova, M. Vocka, J. Spacek, L. Petruzelka, B. Konopasek, P. Dundr, H. Skalova, K. Nemejcova, G. Botti, F. Tatangelo, P. Delrio, G. Ciliberto, M. Maio, L. Laghi, F. Grizzi, T. Fredriksen, B. Buttard, M. Angelova, A. Vasaturo, P. Maby, S.E. Church, H.K. Angell, L. Lafontaine, D. Bruni, C. El Sissy, N. Haicheur, A. Kirilovsky, A. Berger, C. Lagorce, J.P. Meyers, C. Paustian, Z. Feng, C. Ballesteros-Merino, J. Dijkstra, C. van de Water, S. van Lent-van Vliet, N. Knijn, A.-M. Mușină, D.-V. Scripcariu, B. Popivanova, M. Xu, T. Fujita, S. Hazama, N. Suzuki, H. Nagano, K. Okuno, T. Torigoe, N. Sato, T. Furuhata, I. Takemasa, K. Itoh, P.S. Patel, H.H. Vora, B. Shah, J.B. Patel, K.N. Rajvik, S.J. Pandya, S.N. Shukla, Y. Wang, G. Zhang, Y. Kawakami, F.M. Marincola, P.A. Ascierto, D.J. Sargent, B.A. Fox, J. Galon, International validation of the consensus Immunoscore for the classification of colon cancer: a prognostic and accuracy study., Lancet. 391 (2018) 2128–2139. https://doi.org/10.1016/S0140-6736(18)30789-X.](https://sciwheel.com/work/bibliography/5331249)
26. [L.M. Coussens, L. Zitvogel, A.K. Palucka, Neutralizing tumor-promoting chronic inflammation: a magic bullet?, Science. 339 (2013) 286–291. https://doi.org/10.1126/science.1232227.](https://sciwheel.com/work/bibliography/636918)
27. [D.G. DeNardo, B. Ruffell, Macrophages as regulators of tumour immunity and immunotherapy., Nat. Rev. Immunol. 19 (2019) 369–382. https://doi.org/10.1038/s41577-019-0127-6.](https://sciwheel.com/work/bibliography/6367684)
28. [Y. Fong, J. Fortner, R.L. Sun, M.F. Brennan, L.H. Blumgart, Clinical score for predicting recurrence after hepatic resection for metastatic colorectal cancer: analysis of 1001 consecutive cases., Ann. Surg. 230 (1999) 309–18; discussion 318. https://doi.org/10.1097/00000658-199909000-00004.](https://sciwheel.com/work/bibliography/697513)
29. [N. A-Gonzalez, J.A. Quintana, S. García-Silva, M. Mazariegos, A. González de la Aleja, J.A. Nicolás-Ávila, W. Walter, J.M. Adrover, G. Crainiciuc, V.K. Kuchroo, C.V. Rothlin, H. Peinado, A. Castrillo, M. Ricote, A. Hidalgo, Phagocytosis imprints heterogeneity in tissue-resident macrophages., J. Exp. Med. 214 (2017) 1281–1296. https://doi.org/10.1084/jem.20161375.](https://sciwheel.com/work/bibliography/3528097)

**Figure Legends**

**Figure 1 TAMs morphology: assessment**

Starting from 2 um-thick slides stained with the anti-CD163 antibody (a), the metastasis tumoral tissue was defined, the zone of the invasive tumor margin in the peritumor tissue was located (b) and area and perimeter of macrophages were annotated by manually tracing their outlines (c).

*T, tumor tissue; IM, Invasive Margin; PT Peritumor Tissue.*

**Figure 2 Univariate analysis, association between TAMs morphology and Disease-Free Survival**

A receiver operating characteristic curve was created to define optimal cut-off point for the prediction of recurrence after hepatectomy (a) and, after univariate analysis (b), macrophage area confirmed a statistically significant association with DFS (p=0.0002), with smaller macrophages conferring a more favorable prognosis.

*DFS, Disease-Free Survival*

**Figure 3 Density maps of TAMs**

(a). Density maps showing the distribution of S-TAMs and L-TAMs on a 500 μm wide peritumor area that run on the whole contour of the tumor, in a subset of 12 patients classified as “early recurrence” (recurrence within three months after surgery) or “no recurrence” (no recurrence after 48 months from surgery). (b). Histogram showing total area of the high-density zones of L-TAMs in each slide (defined *foci* of L-TAMs.) in the two groups of patients (P=0.02),

*TAMs, Tumor Associated Macrophages; S-TAMs, Small TAMs; L-TAMs, Large TAMs*

**Tables**

**Table 1 Description of the series**

| **Demographics, tumoral, chemotherapy, and post-operative details** | | | | |
| --- | --- | --- | --- | --- |
| Gender, M/F, n (%) | 60 (71) / 24 (29) |  | Neoadjuvant chemotherapy, n (%) | 59 (70) |
| Age, yr, median (range) | 62 (36-78) |  | Chemotherapy lines, median (range) | 1 (1-2) |
| Size of CLMs cm, median (range) | 1,9 (0–11) |  | Type of chemotherapy, n (%) |  |
| Number of CLMs, median (range) | 2 (1-28) |  | 5FU Based | 36 (43) |
| Bilobar disease, n (%) | 35 (42) |  | Oxaliplatin based | 42 (50) |
| Preoperative CEA, median (range) | 4 (0.5-520.6) |  | Irinotecan based | 33 (39) |
| Preoperative Ca 19.9, median (range) | 13.45 (0.6-614.3) |  | +Anti-VEGF | 18 (21) |
| Grading of the primary tumor, G3-4, n (%) | 13 (15) |  | +Anti-EGFR | 33 (39) |
| Staging of the primary tumor |  |  | RAS, mutated, n (%) | 27 (32) |
| T status, T3-4, n (%) | 64 (76) |  | Order of resection: Bowel first, n (%) | 21 (45) |
| N status, N+, n (%) | 50 (60) |  | Liver-First, n (%) | 12 (25) |
| Synchronous presentation, n (%) | 47 (56) |  | Simultaneous resection, n (%) | 14 (30) |
| Site of primary tumor, |  |  | Overall post-operative complications, n (%) | 19 (23) |
| Colon, n (%) | 59 (70) |  | Major complications, (Clavien-Dindo 3b or more), n (%) | 5 (6) |
| Rectum, n (%) | 27 (32) |  | 90-day post-operative mortality, n (%) | 0 (0) |
| *CLM, Colorectal Liver Metastases; CEA, Carcinoembryonic antigen; 5-FU, Fluorouracil; VEGF, Vascular Endothelial Growth Factor; EGFR, Epidermal Growth Factor Receptor* | | | | |

**Table 2 Univariate analysis**

|  | **No recurrence** | **Recurrence** | **p** |
| --- | --- | --- | --- |
| **n (%)** | **18 (28)** | **66 (79)** |  |
| Age, years, median (range) | 58.5 (50-70) | 62 (36-78) | ns |
| Sex, male, n (%) | 12 (60) | 49 (74) | ns |
| Tumor Number, n, median (range) | 1 (1-28) | 2 (1-23) | ns |
| Max size, mm, median (range) | 17 (1-45) | 20 (1-110) | ns |
| Neoadjuvant chemotherapy, n (%) | 8 (44) | 51 (77) | *<0.01* |
| CEA elevated, n (%) | 11 (61) | 42 (64) | ns |
| Ca 19.9 elevated, n (%) | 1 (6) | 13 (20) | ns |
| T3-4, n (%) | 12 (67) | 52 (79) | ns |
| N+, n (%) | 5 (28) | 45 (68) | *<0.01* |
| Synchronous presentation, n (%) | 6 (33) | 41 (62) | *0.03* |
| G3-4, n (%) | 2 (11) | 11 (17) | ns |
| kRAS, mutated, n (%) | 2 (11) | 23 (35) | ns |
| Bilobar disease, n (%) | 7 (39) | 27 (41) | ns |
| Primary site, rectum, n (%) | 5 (28) | 22 (33) | ns |
| TAM area | 150.4 (97.1-301.1) | 217.0 (98.5-388.6) | *<0.01* |
| *TAM, Tumor Associated Macrophages; CEA, Carcinoembryonic antigen* |  |  |  |
|  | | | |

**Table 3 Multivariate analysis**

| **Factor** | **COX model** | | |
| --- | --- | --- | --- |
|  | **HR** | **95% CI** | **P value** |
| TAMs area; L-TAMs vs S-TAMs | 5.04 | 1.70 - 14.94 | *0.003* |
| Neoadjuvant chemotherapy, yes vs no | 1.27 | 0.68 - 2.37 | 0.453 |
| N of the primary tumor, positive vs negative | 1.46 | 0.84 - 2.55 | 0.181 |
| Synchronous presentation yes vs no | 1.08 | 0.63 - 1.84 | 0.786 |
| *TAMs, Tumor Associated Macrophages; S-TAMs, Small TAMs; L-TAMs, Large TAMs* | | | |

**Table S1 Multivariate analysis (Overall Survival)**

| **Factor** | **COX model** | | |
| --- | --- | --- | --- |
|  | **HR** | **95% CI** | **P value** |
| TAMs area; L-TAMs vs S-TAMs | 1.13 | 0.30-4.29 | 0.853 |
| Neoadjuvant chemotherapy, yes vs no | 1.61 | 0.63-4.12 | 0.674 |
| Age | 1.01 | 0.97-1.04 | 0.32 |
| Ca19.9 greater than 40 IU/L | 3.33 | 1.37-8.10 | *<0.01* |
| Bilobar disease yes vs no | 2.62 | 1.23-5.56 | *0.01* |
| *TAMs, Tumor Associated Macrophages; S-TAMs, Small TAMs; L-TAMs, Large TAMs* | | | |
